# A Rare Case of Sarcoidosis Involving Male Breast Tissue

**DOI:** 10.7759/cureus.21387

**Published:** 2022-01-18

**Authors:** John Grove, Casey Meier, Bahaaeldin Youssef, Patrick Costello

**Affiliations:** 1 College of Osteopathic Medicine, Lincoln Memorial University DeBusk College of Osteopathic Medicine, Knoxville, USA; 2 Pathology, East Tennessee State University Quillen College of Medicine, Johnson City, USA

**Keywords:** biopsy, mammogram, granulomas, bilateral breast masses, sarcoidosis

## Abstract

Sarcoidosis is a multisystem, inflammatory granulomatous disease that rarely involves breast tissue. The pathophysiology of this chronic granulomatous condition is not well understood but is thought to be multifactorial, involving environmental influences causing an amplified immune response. A key histomorphology feature in sarcoidosis is the presence of non-necrotizing granulomas. In this case, we report a 41-year-old African-American man with a known history of sarcoidosis of the lung who presented with gynecomastia and bilateral breast tenderness with palpable nodules. Subsequent biopsy and microscopic examination of the breast nodules revealed diffuse involvement with non-necrotizing granulomas in both breasts. A final diagnosis of extensive sarcoidosis involving breast tissue was rendered after excluding other causes of non-necrotizing granulomas. The patient underwent a bilateral mastectomy to remove the breast nodules. This case discusses sarcoidosis involving an unusual site.

## Introduction

Sarcoidosis is a multisystem inflammatory disease characterized by the presence of non-necrotizing granulomas in one or more sites, predominantly consisting of epithelioid histiocytes and T lymphocytes [1,2]. Ninety percent (90%) of sarcoidosis present with hilar lymphadenopathy are detected radiologically as bilateral lung opacities [3-5]. In some cases, granulomas can also be seen in multiple areas such as the skin, eyes, joints, kidneys, and other organs [3]. The pathophysiology of sarcoidosis is not well understood; however, the etiology is attributed to a combination of genetic and environmental factors such as beryllium or other dust particles that trigger an amplified immune reaction [1,3]. 

Sarcoidosis is known to primarily affect young to middle-aged adults with the highest incidence among the African-American population (34 per 100,000 individuals) compared to Caucasians [1]. Women have a slightly higher prevalence of sarcoidosis (1.3%) compared to men (1%) [3]. Here we report a 41-year-old African-American man with biopsy-confirmed bilateral sarcoidosis of the breast who was treated with bilateral mastectomy.

## Case presentation

A 41-year-old African-American man presented with bilateral breast tenderness and slowly enlarging bilateral breast nodules that started five months before he came in. His past medical history was significant for Bell’s palsy, Herpes zoster encephalitis, and sarcoidosis of the lung. He denied smoking or tobacco use. There was no family history of sarcoidosis, any autoimmune condition, or breast cancer. 

Physical examination revealed bilateral breast masses, each formed by multiple firm nodules. The patient denied shortness of breath, cough, fatigue, fever, chills, and rash. He did not have any significant weight loss or enlarged lymph nodes. 

**Figure 1:**
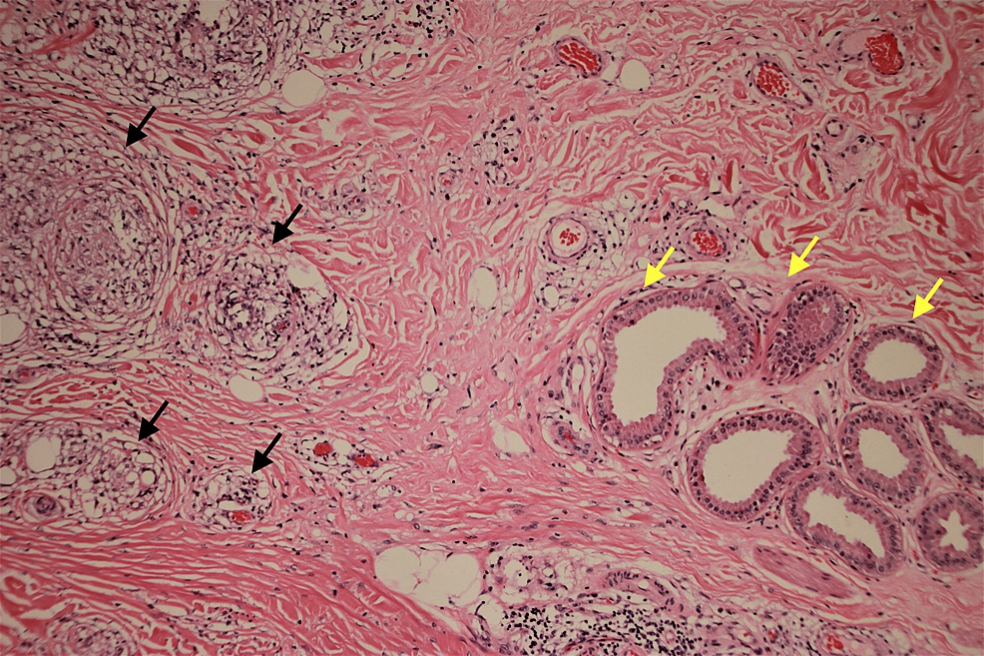
Histologic section showing non-necrotizing granulomas Histologic section showing non-necrotizing granulomas (black arrows) adjacent to breast ductules with apocrine metaplasia (yellow arrows). Hematoxylin and Eosin, 40x

**Figure 2:**
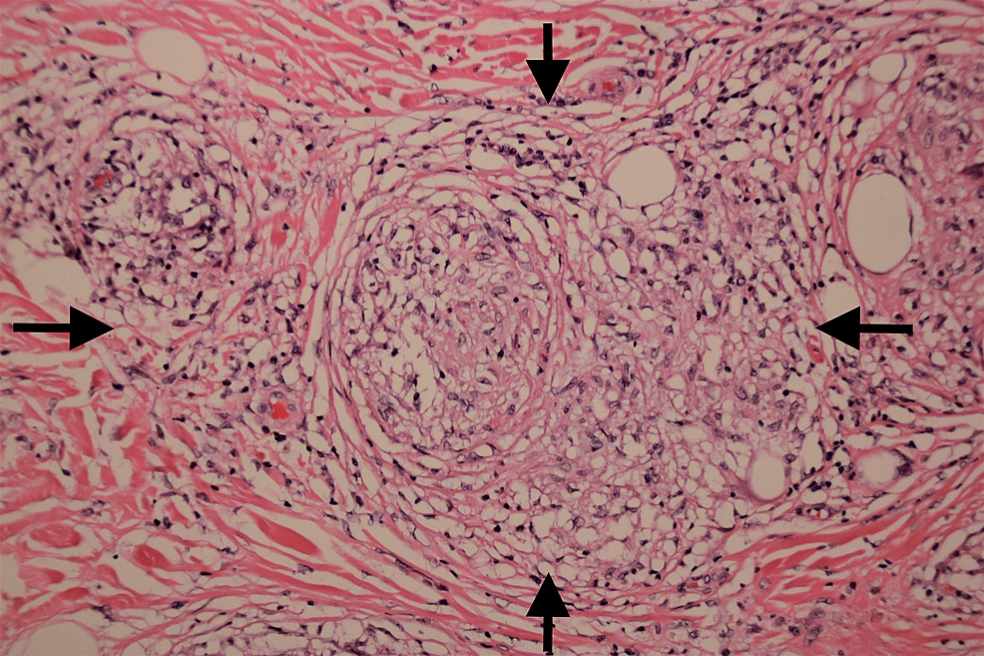
High power image of non-necrotizing granuloma High power image of a non-necrotizing granuloma (black arrows) surrounded by hyaline type fibrosis. Hematoxylin and Eosin, 100x.

Craniocaudal (CC) and mediolateral mammography (MLO) demonstrated heterogeneous microcalcifications in the left upper breast quadrant and right retroareolar region with a moderate flame-shaped increased density. Breast ultrasonography showed an additional macrocalcification in the upper inner quadrant of the right breast, approximately 0.6 cm x 0.6 cm x 0.6 cm. No suspicious adenopathy was identified in either axillary region. Due to raised clinical concern for the calcifications, a subsequent breast biopsy was performed. Microscopic examination revealed bilateral non-necrotizing pauci-inflammatory granulomas composed of epithelioid histiocytes, macrophages, and lymphocytes (Figures 1, 2). The presence of microcalcifications was also possible. Acid-fast bacteria and Grocott's methenamine silver stains showed no appearance of fungi or mycobacteria. No evidence of in situ carcinoma or malignancy was noted. Bilateral mastectomy was performed to yield 7.5 cm (left) and 5.5 cm (right) masses of benign fibro-adipose tissue that showed the same histomorphology with negative acid-fast bacteria and Grocott's methenamine silver stains. The masses were completely excised with no in situ or invasive carcinoma detected. The patient’s presentation supported the diagnosis of sarcoidosis involving the breast tissue. A determination of sarcoidosis in the breast tissue was made in the clinical context of a previous diagnosis of sarcoidosis.

## Discussion

Sarcoidosis is a chronic granulomatous inflammatory disease that primarily affects the lungs and surrounding lymph nodes [1]. Extrapulmonary locations of sarcoidosis include skin, eyes, kidney, heart, central nervous system, and breast tissue on rare occasions [1]. Clinical manifestations of pulmonary involvement typically include shortness of breath, dry cough, palpable lymph nodes, and fatigue [1]. Sarcoidosis is characterized histologically by the presence of non-necrotizing granulomas. Other microscopic features that may help identify sarcoidosis include laminated masses of calcium and protein, otherwise known as Schaumann bodies, and stellate inclusions in giant cells, termed asteroid bodies [2]. Sarcoidosis primarily affects those ranging from 20-40 years of age, with the highest prevalence seen in African-American women [6]. Sarcoidosis can be detected as architectural changes on mammography, MRI, and ultrasound studies; however, a biopsy is required to make the diagnosis [7]. In breast sarcoidosis imaging frequently mimics breast cancer that must be ruled out with biopsy [8]. Sarcoidosis limited to breast tissue is a benign process that can be treated surgically, as in our reported case, bilateral mastectomy was the primary treatment.

A few cases reported breast involvement in sarcoidosis [8-12]. However, in a review of 25 case reports of breast sarcoidosis, all were women [8]. Sarcoidosis can mimic breast cancer, and there are documented breast cancer cases in patients with sarcoidosis; thus, it is crucial to exclude in situ or invasive carcinoma through histopathologic examination [13,14]. Treatment involves removing inflammatory non-necrotizing granulomatous tissue with or without steroid treatment and regular follow-up [8]. In this study, we reported a man presenting with bilateral breast tenderness with palpable nodules identified as sarcoidosis by biopsy. Bilateral mastectomy was curative.

## Conclusions

An African-American male presenting with breast masses is typically alarming for cancer. Here we report the rare case of sarcoidosis involving bilateral breast tissue in a man. Further reporting may reveal more instances of this unique occurrence.
